# Comparative anti-inflammatory and lipid-normalizing effects of metformin and omega-3 fatty acids through modulation of transcription factors in diabetic rats

**DOI:** 10.1186/s12263-016-0518-4

**Published:** 2016-03-17

**Authors:** Abhijit Ghadge, Abhay Harsulkar, Manjiri Karandikar, Vijaya Pandit, Aniket Kuvalekar

**Affiliations:** 1 Nutrigenomics and Functional Foods Laboratory, Interactive Research School for Health Affairs (IRSHA), Bharati Vidyapeeth Deemed University, Pune-Satara Road, Pune, Maharashtra 411043 India; 2 Department of Pathology, Bharati Vidyapeeth Medical College, Bharati Vidyapeeth Deemed University, Pune-Satara Road, Pune, Maharashtra 411043 India; 3 Department of Pharmacology, Bharati Vidyapeeth Medical College, Bharati Vidyapeeth Deemed University, Pune-Satara Road, Pune, Maharashtra 411043 India

**Keywords:** Fish oil, Flax oil, Metformin, Omega-3 fatty acids, Streptozotocin

## Abstract

**Background:**

Emerging evidence suggests beneficial effects of omega-3 fatty acids on diabetic complications. The present study compared the progressive effects of metformin and flax/fish oil on lipid metabolism, inflammatory markers, and liver and renal function test markers in streptozotocin-nicotinamide-induced diabetic rats.

**Methods:**

Streptozotocin-induced diabetic rats were randomized into control and four diabetic groups: streptozotocin (STZ), metformin (200 mg/kg body weight (b.w)/day (D)), flax and fish oil (500 mg/kg b.w/D).

**Results:**

Metformin and flax and fish oil exhibited increased expression of transcription factor peroxisome proliferator-activated receptor γ while the treatment downregulated sterol regulatory element-binding protein 1 and nuclear factor kβ as compared to those of the STZ group. Apart from modulation of transcription factor expression, the expression of fatty acid synthase, long chain acyl CoA synthase, and malonyl-CoA-acyl carrier protein transacylase was lowered by flax/fish oil treatment. Serum cholesterol, triglycerides, and VLDL were also significantly reduced in the treatment groups as compared to those in the STZ group. Although pathological abnormalities were seen in the liver and kidneys of rats on metformin, no significant changes in liver/renal function markers were observed at day 15 and day 30 of the treatment groups. Flax/fish oil had protective effects toward pathological abnormalities in the liver and kidney. Flax/fish oil improved lipid profile and alkaline phosphatase at day 30 as compared to that at day 15.

**Conclusions:**

The present study demonstrates potential beneficial effects of metformin and flax/fish oil intervention in improving serum lipid profile by regulating the expression of transcription factors and genes involved in lipid metabolism in diabetic rats. In addition, these interventions also lowered the expression of atherogenic cytokines. The protective effects of flax/fish oil are worth investigating in human subjects on metformin monotherapy.

## Background

Omega-3 polyunsaturated fatty acids (n-3 PUFA) constitute a fundamental part of cell membranes and exhibit a diverse range of membrane functions including functioning of transporters, signal transduction pathways, and gene expression (Liu and Ma 2014; Ander et al. 2003). Alpha-linolenic acid (ALA; 18:3 n-3) is a short chain n-3 PUFA obtained from flaxseed (a plant-derived food item) (Dessì et al. 2013; Mozaffarian and Wu 2012; Connor 2000) while eicosapentaenoic acid (EPA) and docosahexaenoic acid (DHA; 22:6 n-3) are the long chain n-3 PUFA derived from a seafood source, fish oil (Swanson et al. 2012). There is strong scientific evidence demonstrating anti-inflammatory, anti-atherogenic, vasodilatory, and lipid-lowering properties of n-3 PUFA (Jangale et al. 2013; Mozaffarian and Wu 2012; Connor 2000) due to which they have been implicated in some chronic diseases like cardiovascular disease (Ander et al. 2003), diabetes (Wu et al. 2012), and autoimmune diseases (Calder 2007). In view of this, dietary n-3 PUFA are considered as significant nutrients involved in metabolic regulation. There is a recent surge in research on effects of functional foods, like n-3 PUFA for the management of type 2 diabetes mellitus (T2DM).

Diabetes consists of a group of metabolic disorders involving distinct pathogenic mechanisms with hyperglycemia, arising due to impaired metabolism of glucose, lipids, and proteins (Prabhakar et al. 2013). T2DM is frequently associated with abnormal lipid profile (Ozder 2014) with perturbations in the lipid metabolism affected by abnormalities in the expression of hepatic transcription factors and genes (Erejuwa et al. 2012). Moreover, diabetics are more prone to develop chronic complications related to cardiovascular, renal (Forbes and Cooper 2013), and peripheral vascular tissues (King 2008).

Several oral hypoglycemic drugs such as biguanides, sulfonylurea, and thiazolidenediones are commonly used for the treatment of T2DM. Metformin is an old and widely used first-line agent, known for its anti-hyperglycemic properties, and is also reported to improve lipid profile, fat redistribution (Rojas and Gomes 2013), and chronic liver diseases (Zheng et al. 2015) and lower microvascular and macrovascular complications associated with T2DM (Kooy et al. 2009). Though it has rarely been reported to induce hepatocellular and cholestatic hepatic injury (Saadi et al. 2013) and hepatotoxicity (Miralles-Linares et al. 2012), there are very few reports examining its long-term effects for the management of T2DM.

Some investigations have evaluated the individual effects of metformin (Wang et al. 2014) or n-3 PUFA on the regulation of lipid metabolism genes in diabetes (Devarshi et al. 2013). The lipid-lowering actions of n-3 PUFA are attributed to the regulation of key transcription factors like peroxisome proliferator-activated receptors (PPAR) and sterol regulatory element-binding protein (SREBP), that control hepatic lipid metabolism (Di Minno et al. 2012). However, there are some conflicting results stating that supplementation with fish oil does not delay the onset of diabetes in rats at 12 months of age (Cummings et al. 2010) while other reports state that n-3 PUFA slows the progression of T2DM and its complications (Nettleton and Katz 2005). However, there is lack of evidence for these effects of n-3 PUFA in T2DM and the underlying molecular mechanisms have not been well evaluated (Devarshi et al. 2013; Wu et al. 2012). A review by Hendrich points a need to carry out high-quality studies to assess the effects of ALA (Hendrich 2010). The above reports suggest the need for a meticulous evaluation of effects of ALA, EPA, and DHA on lipid metabolism and its underlying mechanism.

It is well known that streptozotocin (STZ) has a selective cytotoxic action on β-cells in the islets of Langerhans whereas nicotinamide has a partial protective role on β-cells against STZ (Szkudelski 2012) and is commonly used for induction of type 2 diabetes mellitus in experimental animals (Devarshi et al. 2013; Pari and Saravanan 2007; Saravanan and Pari 2007; Brenna et al. 2003). Additionally, STZ-nicotinamide is considered as a suitable T2DM model to study the effects of metformin as well as herbal (Maheshwari et al. 2014; Sharma et al. 2012; Mohammadi et al. 2012; Li et al. 2011) and nutritional (Devarshi et al. 2013; Jangale et al. 2013) interventions as indicated by several animal studies. We selected peripheral blood mononuclear cells (PBMCs) to study gene expression profile since the blood is the most accessible tissue in humans, and as the profile of multiple tissues is shared with PBMCs, they reflect important metabolic changes in the liver (Konieczna et al. 2014). However, there are no reports examining the temporal effects of metformin and flax/fish oil diets on the lipid metabolism and biochemical markers in T2DM. Moreover, potential of these treatments to affect duration of the disease is poorly understood.

We hypothesized that metformin and omega-3 fatty acids could improve lipid metabolism and inflammatory cytokines by modulating the expression of transcription factors in STZ-nicotinamide-induced diabetic rats. The objectives of the present study were to examine the comparative effects of metformin and omega-3 fatty acids on serum lipid profile, expression of transcription factors and genes involved in lipid metabolism, and inflammation in STZ-nicotinamide-induced diabetic rats. Additionally, the progressive effects of these treatments on liver/renal function test markers and histological changes in these tissues were evaluated. We also compared the effects of these treatments on the above parameters at post treatment day 15 (D15) and day 30 (D30) to study their effects on the duration of disease.

## Methods

### Chemicals and reagents

Flax oil (Alvel-500) capsules were purchased from Real World Nutritional Laboratory (Pune, India) that contained 50 % alpha-linolenic acid (ALA), 20 % oleic acid, and 12 % linoleic acid. Fish oil capsules (Maxepa) were purchased from Merck Limited (Goa, India) which contained 60 % EPA and 40 % DHA. Streptozotocin (STZ), nicotinamide (Sigma Life Sciences, USA), and metformin (Glycomet 250 mg; USV Limited) were purchased from a local pharmacy.

### Experimental animals

The study was carried out as per the CPCSEA (Committee for the Purpose of Control and Supervision of Experiments on Animals) guidelines after the approval of Institutional Animal Ethics Committee (Ref. No: BVDUMC/189/2014-2015). The male albino Wistar rats weighing between 150 and 200 g were procured from the institutional animal house and maintained under standard conditions throughout the experimentation (temperature 25 ± 2 °C, 12-h light:12-h dark cycle). Animals were fed with standard pellet diet (Nutrivet life science, Pune, M.S., India), and water was supplied ad libitum.

### Diabetes induction

**Fig. 1:**
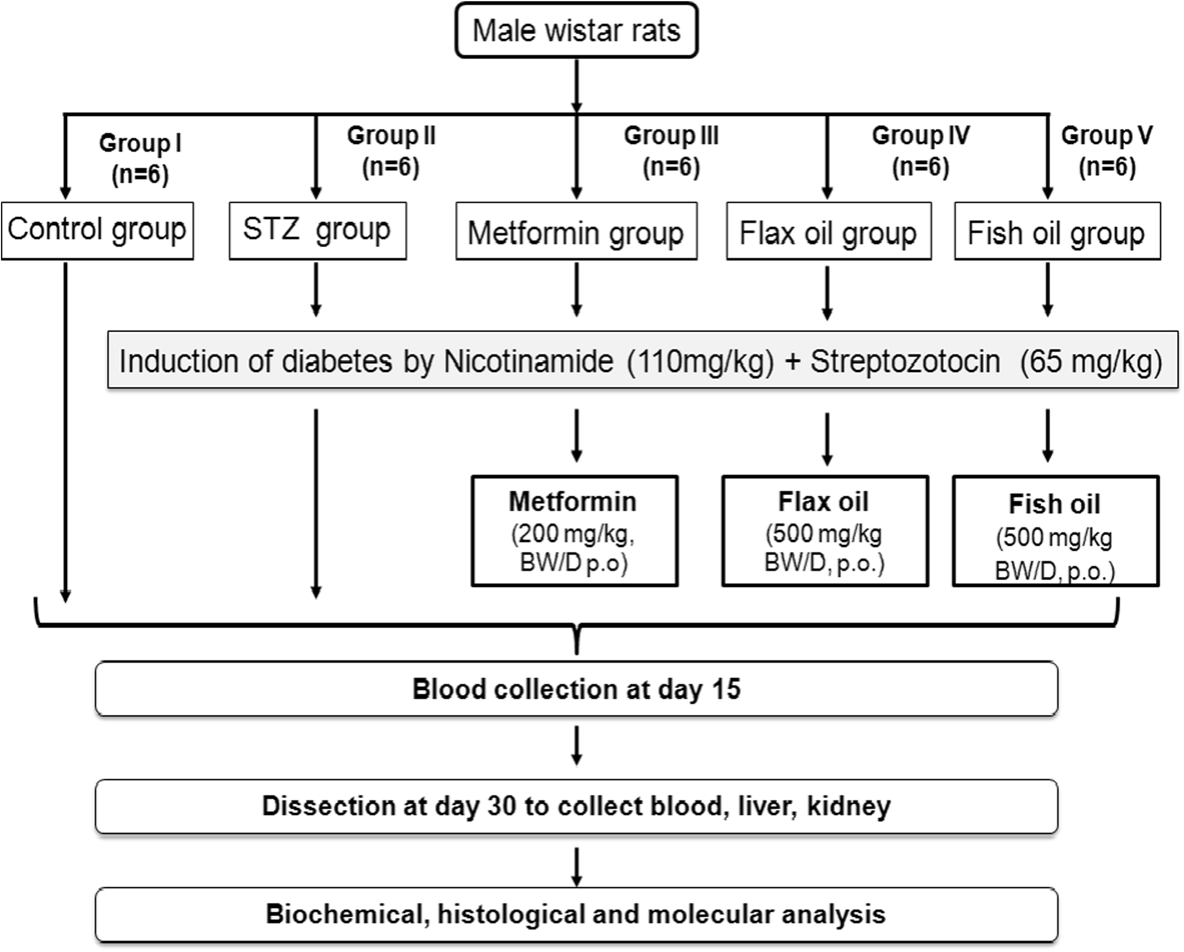
Study design

Animals were randomly assigned to five groups (*n* = 6). Diabetes was induced by administration of nicotinamide (110 mg/kg body weight) in saline through intra-peritoneal (i.p) injection. After 15 min, STZ was administered (65 mg/kg body weight i.p) in sodium citrate buffer, pH 4.0. The fasting blood glucose levels of the rats were estimated after 48 h and on day 7 of STZ injection. Stable hyperglycemia was confirmed by elevated fasting blood glucose levels on day 7 after STZ injection. Animals with fasting blood glucose levels above 200 mg/dl were considered as diabetic and used for the study. After development of stable hyperglycemia, metformin or flax/fish oil treatment was given for 30 days. The dose of flax and fish oil was based on the earlier study carried out in our laboratory (Chavan et al. 2013). The study protocol is given in Fig. 1. Animals were randomly assigned to one of the following groups: group I: control group (*n* = 6): received feed and water normally throughout the experiment; group II: STZ-induced diabetic group (*n* = 6): administered nicotinamide (110 mg/kg body weight) and STZ (65 mg/kg body weight) i.p single dose; group III: metformin group (*n* = 6): administered metformin (200 mg/kg, body weight/D p.o); group IV: flax oil group (*n* = 6): administered flax oil (500 mg/kg body weight/D, p.o); group V: fish oil group (*n* = 6): administered fish oil (500 mg/kg body weight/D, p.o). After 15 days of treatment, animals were fasted overnight and blood was collected by retro-orbital puncture. Animals were sacrificed after 30 days of treatment. Liver and kidney tissues were excised immediately, washed in saline, weighed, and stored in 10 % neutral buffered formalin for histological analysis. Blood was collected and centrifuged at 2000 rpm for 15 min to collect serum for biochemical estimations.

### Blood biochemistry

Serum glucose, total cholesterol, triglyceride, high-density lipoprotein (HDL), low-density lipoprotein (LDL), serum glutamic oxaloacetic transaminase (SGOT), serum glutamic pyruvic transaminase (SGPT) and alkaline phosphatase (ALP), total bilirubin, creatinine, urea, albumin, and total protein were estimated using commercial kits (Coral Clinical Systems, Goa, India). Very low-density lipoprotein cholesterol (VLDL) was estimated by using the formula: (triglyceride/5).

### Quantitative real-time reverse transcription-polymerase chain reaction analysis

PBMCs were isolated by density gradient centrifugation by layering the blood sample over Histopaque-1077 (Sigma-Aldrich, Inc., USA). RNA was isolated by TRIzol (Invitrogen Co., Carlsbad, CA, USA) method. Total RNA was quantified using NanoDrop (ND1000, USA). The quality of RNA was analyzed by agarose gel electrophoresis. Complementary DNA (cDNA) was prepared by using high-capacity cDNA Reverse Transcription kit (Applied Biosystems). Standard quantitative real-time transcription-polymerase chain reaction (qRT-PCR) was performed with the use of SYBR Green master mix (Applied Biosystems) for the following genes: PPARγ, SREBP1, nuclear factor kappa β (NFκβ), fatty acid synthase (FAS), long chain acyl CoA synthetases (ACSL), malonyl-CoA-acyl carrier protein transacylase (MCAT), tumor necrosis factor α (TNFα). KicqStart Primers (Sigma, USA) were used for gene expression studies and are listed in Table 1. qRT-PCR was performed using the Applied Biosystems 7300 Standard system. The reactions for each gene were performed in duplicate. Relative expression levels of genes were calculated and expressed as 2^ΔCT^ where ΔCT is *C*_T_ (GAPDH)–*C*_T_ (target gene) and this method is a modification of ${2}^{-\varDelta \varDelta {C}_{\mathrm{T}}}$ method (Gaines et al. 2010).

**Table 1:** List of primers used for quantitative real-time PCR

Gene	Primer name	Sequence (5′−3′)
GAPDH	GAPDH F	AGTTCAACGGCACAGTCAAG
	GAPDH R	TACTCAGCACCAGCATCACC
PPARγ	PPARγ F	AAGACAACAGACAAATCACC
	PPARγ R	CAGGGATATTTTTGGCATACTC
SREBP1	SREBP F	AAACCTGAAGTGGTAGAAAC
	SREBP R	TTATCCTCAAAGGCTGGG
NFKB	NFKB F	AAAAACGAGCCTAGAGATTG
	NFKB R	ACATCCTCTTCCTTGTCTTC
FAS	FAS F	AAAAGGAAAGTAGAGTGTGC
	FAS R	GACACATTCTGTTCACTACAG
ACSL	ACSL F	ACATTATGAACGATTGCTCC
	ACSL R	GCATTACACACTCTACAACG
MCAT	MCAT F	AAAACTCTAGGCTCAATCAAC
	MCAT R	GGATGTGTGTATTTATGCCC
TNFα	TNFα F	CTCACACTCAGATCATCTTC
	TNFα R	GAGAACCTGGGAGTAGATAAG

*GAPDH* glyceraldehyde-3-phosphate dehydrogenase, *PPARγ* peroxisome proliferator-activated receptors γ, *SREBP1* sterol regulatory element-binding protein 1, *NFκβ* nuclear factor kappa β, *FAS* fatty acid synthase, *ACSL* long chain acyl CoA synthetases, *MCAT* malonyl-CoA-acyl carrier protein transacylase, *TNFα* tumor necrosis factor α (Make: Sigma-Aldrich; *F* forward primer sequence, *R* reverse primer sequence)

### Histological analysis

Paraffin-embedded liver and kidney tissues were cut at 4 μm and stained with hematoxylin and eosin. The slides were examined under a binocular microscope (Make: Olympus IX71) and photographed by using Image Pro Plus (v5.1.2.59).

### Statistical analysis

Results are presented as mean ± standard error (SE). All the statistical analyses were performed using SPSS PC+ package (Version 20, Chicago, IL, USA). The data were checked for normal distribution by testing for skewness. Skewed variables were transformed to normality using log to the base 10 transformation. Statistical differences between means in different groups were determined using one-way analysis of variance (ANOVA) test followed by post hoc Bonferonni multiple correction test. Mean values of various parameters from each group at D15 were compared with those at D30 using Student’s *t* test. *P* ≤ 0.05 was considered statistically significant.

## Results

### Food intake, body weights, and organ weights

The food intake of animals from the STZ group was higher at D1 (*p* < 0.01), D15, and D30 (*p* < 0.05) as compared to that from the control. The body weights were lower at D15 (*p* < 0.05) and D30 (*p* < 0.01) when compared with those of the control (Table 2). Liver weights, hepatic index, and kidney weights were comparable while kidney index (*p* < 0.01) was higher in the STZ group as compared to that in the control (Table 2).

**Table 2:** Food intake and body, liver, and kidney weights of animals

	Control (*n* = 6)	STZ (*n* = 6)	Metformin (*n* = 6)	Flax oil (*n* = 6)	Fish oil (*n* = 6)
Food intake (g)					
Day 1	14.66 ± 3.58	30.83 ± 0.21**	33.66 ± 0.58	31.83 ± 2.69	30.00 ± 0.65
Day 15	12.66 ± 2.41	23.33 ± 2.07*	27.00 ± 1.27	28.33 ± 1.80	16.66 ± 2.59
Day 30	16.00 ± 2.22	26.50 ± 3.55*	27.00 ± 1.89	33.66 ± 0.45	31.50 ± 1.93
Body weights (g)					
Day 1	236.50 ± 3.48	235.50 ± 8.54	235.50 ± 13.91	245.83 ± 3.84	243.16 ± 15.15
Day 15	292.16 ± 6.55	232.33 ± 20.28*	201.33 ± 14.02	231.83 ± 12.68	202.50 ± 11.64
Day 30	345.50 ± 10.47	223.33 ± 30.34**	176.50 ± 14.93	211.33 ± 13.90	198.33 ± 11.47
Liver and kidney weights					
Liver weight (g)	9.97 ± 0.60	7.68 ± 0.59	6.60 ± 0.50	7.60 ± 0.51	8.24 ± 0.59
Liver index (%)	2.87 ± 0.12	3.60 ± 0.31	3.78 ± 0.19	3.62 ± 0.20	4.17 ± 0.24
Kidney weight (g)	1.60 ± 0.13	1.89 ± 0.08	1.76 ± 0.12	1.91 ± 0.07	1.84 ± 0.03
Kidney index (%)	0.46 ± 0.03	0.90 ± 0.08**	1.01 ± 0.06	0.91 ± 0.04	0.94 ± 0.05

Data are presented as mean ± SE

*p < 0.05, **p < 0.01 for comparison between the control and STZ group

### Serum glucose levels

Serum glucose levels were significantly higher (*p* < 0.01) in the STZ group than those in the control at D15 and D30 (Table 3).

**Table 3:** Serum glucose level and lipid profile at post treatment days 15 and 30

	Control (*n* = 6)	STZ (*n* = 6)	Metformin (*n* = 6)	Flax oil (*n* = 6)	Fish oil (*n* = 6)
Glucose level (mg/dl)					
At day 15	53.72 ± 3.58	294.11 ± 14.85**	340.98 ± 30.14	280.12 ± 12.62	315.36 ± 10.66
At day 30	54.11 ± 4.89	309.65 ± 33.54**	300.64 ± 6.75	315.18 ± 16.20	269.23 ± 16.96
Lipid profile					
Post treatment day 15					
Cholesterol (mg/dl)	43.37 ± 3.76	98.24 ± 12.83**	58.79 ± 4.47^#^	87.31 ± 9.03	69.44 ± 3.16
Triglyceride (mg/dl)	72.74 ± 6.15	171.96 ± 39.49	145.58 ± 20.68	114.01 ± 15.45	133.95 ± 23.67
HDL (mg/dl)	10.03 ± 0.83	22.10 ± 2.11**	8.09 ± 1.55^##^	22.93 ± 1.95	13.80 ± 1.17^#^
LDL (mg/dl)	17.31 ± 1.44	38.12 ± 3.65**	13.96 ± 2.68^##^	39.56 ± 3.37	23.82 ± 2.20^#^
VLDL (mg/dl)	14.54 ± 1.23	34.39 ± 7.89	29.19 ± 4.13	22.80 ± 3.09	26.79 ± 4.73
Post treatment day 30					
Cholesterol (mg/dl)	44.44 ± 2.04	68.22 ± 10.04*	34.35 ± 4.71^##^	49.25 ± 2.82	34.62 ± 4.75^##^
Triglyceride (mg/dl)	66.29 ± 5.96	201.33 ± 31.23**	43.14 ± 8.12^##^	44.81 ± 11.19^##^	42.03 ± 9.13^##^
HDL (mg/dl)	10.71 ± 0.70	9.95 ± 1.10	7.97 ± 1.02	13.05 ± 1.06	8.73 ± 0.71
LDL (mg/dl)	18.48 ± 1.21	17.16 ± 1.90	13.75 ± 1.77	22.52 ± 1.83	15.05 ± 1.23
VLDL (mg/dl)	13.25 ± 1.19	40.26 ± 6.24**	8.62 ± 1.62^##^	8.96 ± 2.23^##^	8.40 ± 1.82^##^

Data are presented as mean ± SE

*HDL* high-density lipoprotein cholesterol, *LDL* low-density lipoprotein cholesterol, *VLDL* very low-density lipoprotein cholesterol

*p < 0.05, **p < 0.01 for comparison between the control and STZ group and ^#^ *p* < 0.05, ^##^ *p* < 0.01 for comparison between the STZ group and treatment groups

### Comparison of glucose levels at D15 and D30 within the groups

Glucose levels in the control, STZ, metformin, and flax oil groups were comparable at D15 and D30. However, glucose levels in the fish oil group were lower (*p* < 0.05) at D30 as compared to those at D15 (Table 5).

### Lipid profile

At D15, the STZ group had higher serum cholesterol (*p* < 0.01), HDL, and LDL levels (*p* < 0.01) than the control group. A higher trend was observed for triglyceride and VLDL (*p* = 0.068) levels in the STZ group than for those in the control group. Animals from the metformin-treated group had lower cholesterol (*p* < 0.05), HDL, and LDL (*p* < 0.01) than those in the STZ group. The fish oil treatment group also lowered HDL and LDL levels (*P* < 0.05) than the STZ group (Table 3).

At D30, higher serum cholesterol (*p* < 0.05), triglyceride, and VLDL levels (*p* < 0.01) were seen in the STZ group than in the control. Metformin group lowered cholesterol, triglyceride, and VLDL (*p* < 0.01) as compared to the STZ group. Flax oil treatment group lowered triglycerides and VLDL levels (*p* < 0.01) than the STZ group. Fish oil treatment group lowered cholesterol, triglyceride, and VLDL (*p* < 0.01) as compared to the STZ group (Table 3).

### Comparison of lipid profile parameters at D15 and D30 within the groups

In the STZ group, serum HDL and LDL levels were lower (*p* < 0.01) at D30 as compared to those at D15. Serum cholesterol, triglyceride, and VLDL levels were lower (*p* < 0.01) at D30 as compared to those at D15 in the metformin group. In the flax and fish oil group, cholesterol, triglyceride, HDL, LDL, and VLDL levels were lower (*p* < 0.01) at D30 as compared to those at D15 (Table 5).

### Expression of genes involved in lipid metabolism

**Fig. 2:**
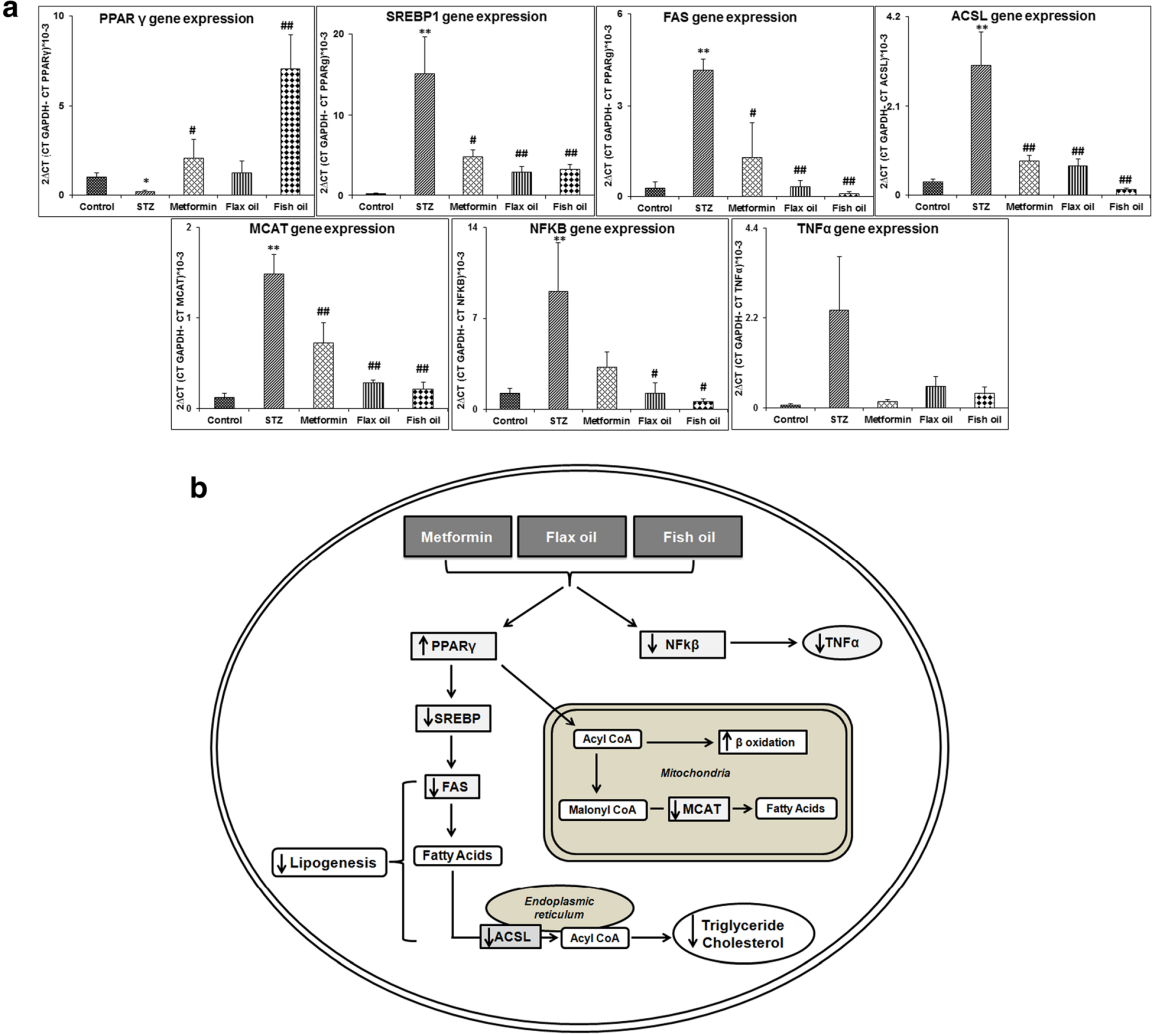
**a** Expression of genes involved in lipid metabolism and inflammation. Data are presented as mean ± SE. *p < 0.05, **p < 0.01 for comparison between the control and STZ group and ^#^ *p* < 0.05, ^##^ *p* < 0.01 for comparison between the STZ group and treatment groups. *GAPDH* glyceraldehyde-3-phosphate dehydrogenase, *PPARγ* peroxisome proliferator-activated receptors γ, *SREBP1* sterol regulatory element-binding protein, *NFκβ* nuclear factor kappa β, *FAS* fatty acid synthase, *ACSL* long chain acyl CoA synthetases, *MCAT* malonyl-CoA-acyl carrier protein transacylase, *TNFα* tumor necrosis factor α. **b** Diagrammatic representation of the possible mechanism of metformin and flax and fish oil on the lipid metabolism and inflammatory cytokines. *PPARγ* peroxisome proliferator-activated receptors γ, *SREBP* sterol regulatory element-binding protein, *NFκβ* nuclear factor kappa β, *FAS* fatty acid synthase, *ACSL* long chain acyl CoA synthetases, *MCAT* malonyl-CoA-acyl carrier protein transacylase, *TNFα* tumor necrosis factor α, *up arrow* up-regulation, *down arrow* down-regulation

The PPARγ gene expression was lower in the STZ group (*p* < 0.05) as compared to that in the control. The treatment with metformin (*p* < 0.05) and fish oil (*p* < 0.01) increased PPARγ expression as compared to that in the STZ group (Fig. 2a).

The SREBP1 gene expression was higher in the STZ group (*p* < 0.01) as compared to that in the control while treatment with metformin (*p* < 0.05) and flax and fish oil lowered (*p* < 0.01) the expression as compared to that in the STZ group (Fig. 2a).

The expression of fatty acid synthase (FAS) was higher in the STZ group (*p* < 0.01) as compared to that in the control whereas the expression was lower in metformin (*p* < 0.05) and flax and fish oil groups (*p* < 0.01) when compared to that in the STZ group (Fig. 2a).

The ACSL gene expression was higher in the STZ group (*p* < 0.01) as compared to that in the control. Metformin and flax and fish oil (*p* < 0.01) lowered the expression of ACSL as compared to that in the STZ group (Fig. 2a).

The MCAT gene expression was higher in the STZ group (*p* < 0.01) as compared to that in the control. Metformin and flax and fish oil (*p* < 0.01) lowered the expression as compared to that in the STZ group (Fig. 2a).

### Expression of genes involved in inflammation

The NFkβ gene expression was higher in the STZ group (*p* < 0.05) as compared to that in the control. The metformin group did not show any significant difference as compared to the STZ group. Flax and fish oil (*p* < 0.05) lowered the expression as compared to that in the STZ group (Fig. 2a).

The TNFα gene expression did not show a significant difference although it was higher in the STZ group than in the control (*p* = 0.123). Metformin (*p* = 0.155) and flax (*p* = 0.404) and fish (*p* = 0.260) oil groups exhibited decreased expression of TNFα as compared to the STZ group, but the decrease was not statistically significant (Fig. 2a).

### Liver function test markers

At D15, serum SGPT and ALP levels were higher (*p* < 0.05) in the STZ group than in the control, but SGOT and bilirubin levels were comparable to those in the control (Table 4).

**Table 4:** Liver and renal function test markers at post treatment days 15 and 30

	Control (*n* = 6)	STZ (*n* = 6)	Metformin (*n* = 6)	Flax oil (*n* = 6)	Fish oil (*n* = 6)
Liver function test markers					
Post treatment day 15					
SGOT (U/ml)	104.10 ± 3.97	123.46 ± 6.98	162.11 ± 20.38	133.84 ± 13.60	116.79 ± 8.20
SGPT (U/ml)	113.08 ± 5.56	167.50 ± 15.28*	199.83 ± 20.03	202.16 ± 7.52	220.08 ± 12.47
ALP (KA Unit)	28.15 ± 4.06	113.46 ± 33.45*	103.88 ± 15.53	91.22 ± 11.19	98.98 ± 4.34
Bilirubin (mg/dl)	0.08 ± 0.18	0.11 ± 0.02	0.12 ± 0.04	0.09 ± 0.01	0.10 ± 0.01
Post treatment day 30					
SGOT (U/ml)	113.71 ± 9.81	124.00 ± 8.45	134.48 ± 9.27	120.38 ± 10.69	136.15 ± 6.41
SGPT (U/ml)	87.50 ± 9.10	134.80 ± 24.83	160.33 ± 32.84	150.00 ± 34.53	198.50 ± 22.43
ALP (KA Unit)	15.78 ± 1.59	75.34 ± 6.49**	69.63 ± 9.41	53.80 ± 5.78	58.29 ± 10.88
Bilirubin (mg/dl)	0.08 ± 0.01	0.11 ± 0.03	0.14 ± 0.01	0.06 ± 0.02	0.06 ± 0.02
Renal function test markers					
Post treatment day 15					
Creatinine (mg/dl)	1.44 ± 0.27	2.38 ± 0.23	1.86 ± 0.17	1.49 ± 0.27	1.50 ± 0.28
Urea (mg/dl)	38.37 ± 3.37	56.11 ± 3.57**	56.88 ± 4.24	46.95 ± 2.26	46.33 ± 3.12
Albumin (g/dl)	2.19 ± 0.15	1.83 ± 0.23	1.61 ± 0.26	1.88 ± 0.11	2.15 ± 0.19
Total protein (g/dl)	4.00 ± 0.18	3.68 ± 0.29	3.86 ± 0.68	3.80 ± 0.25	4.07 ± 0.20
Post treatment day 30					
Creatinine (mg/dl)	1.28 ± 0.15	1.69 ± 0.30	1.61 ± 0.33	1.33 ± 0.10	1.22 ± 0.34
Urea (mg/dl)	40.52 ± 4.50	49.38 ± 3.48	50.88 ± 2.29	60.57 ± 2.35	51.22 ± 4.80
Albumin (g/dl)	2.09 ± 0.14	1.70 ± 0.17	1.54 ± 0.10	1.68 ± 0.11	1.80 ± 0.20
Total protein (g/dl)	3.71 ± 0.09	3.42 ± 0.81	3.08 ± 0.19	3.31 ± 0.14	3.23 ± 0.12

Data are presented as mean ± SE

*SGOT* serum glutamic oxaloacetic transaminase, *SGPT* serum glutamic pyruvic transaminase, *ALP* alkaline phosphatase

*p < 0.05, **p < 0.01 for comparison between the control and STZ group

At D30, serum ALP levels were higher (*p* < 0.01) in the STZ group as compared to those in the control. Metformin and flax and fish oil did not affect liver function test markers at D15 and D30 (Table 4).

### Comparison of liver function test markers between D15 and D30 within the groups

In the control group, serum SGPT and ALP levels were lower (*p* < 0.05) at D15 as compared to those at D30. There was no difference in the liver function test markers in STZ and metformin groups. In both flax oil and fish oil groups, ALP levels were lower (*p* < 0.05) at D30 as compared to those at D15 (Table 5).

**Table 5:** Comparison of biochemical markers between post treatment days 15 and 30 within the groups

	Glucose	Lipid profile					Liver function test markers				Renal function test markers			
		Cholesterol	Triglyceride	HDL	LDL	VLDL	SGOT	SGPT	ALP	Bilirubin	Creatinine	Urea	Albumin	Total protein
*p* value														
Control	0.951	0.833	0.469	0.550	0.550	0.469	0.395	*0.037*	*0.017*	1.000	0.612	0.709	0.638	0.192
STZ	0.662	0.108	0.585	*0.001*	*0.001*	0.586	0.961	0.274	0.310	0.966	0.103	0.215	0.673	0.453
Metformin	0.243	*0.004*	*0.001*	0.950	0.950	*0.001*	0.245	0.333	0.088	0.667	0.522	0.242	0.813	0.301
Flax oil	0.118	*0.007*	*0.005*	*0.001*	*0.001*	*0.005*	0.454	0.170	*0.014*	0.425	0.600	*0.001*	0.272	0.123
Fish oil	*0.044*	*0.000*	*0.005*	*0.004*	*0.004*	*0.005*	0.092	0.425	*0.011*	0.186	0.547	0.413	0.233	*0.004*

*HDL* high-density lipoprotein cholesterol, *LDL* low-density lipoprotein cholesterol, *VLDL* very low-density lipoprotein cholesterol, *SGOT* serum glutamic oxaloacetic transaminase, *SGPT* serum glutamic pyruvic transaminase, *ALP* alkaline phosphatase . The *p* values in italics indicate significant differences in the biochemical markers, within the groups, between post treatment days 15 and 30

### Liver histology

**Fig. 3:**
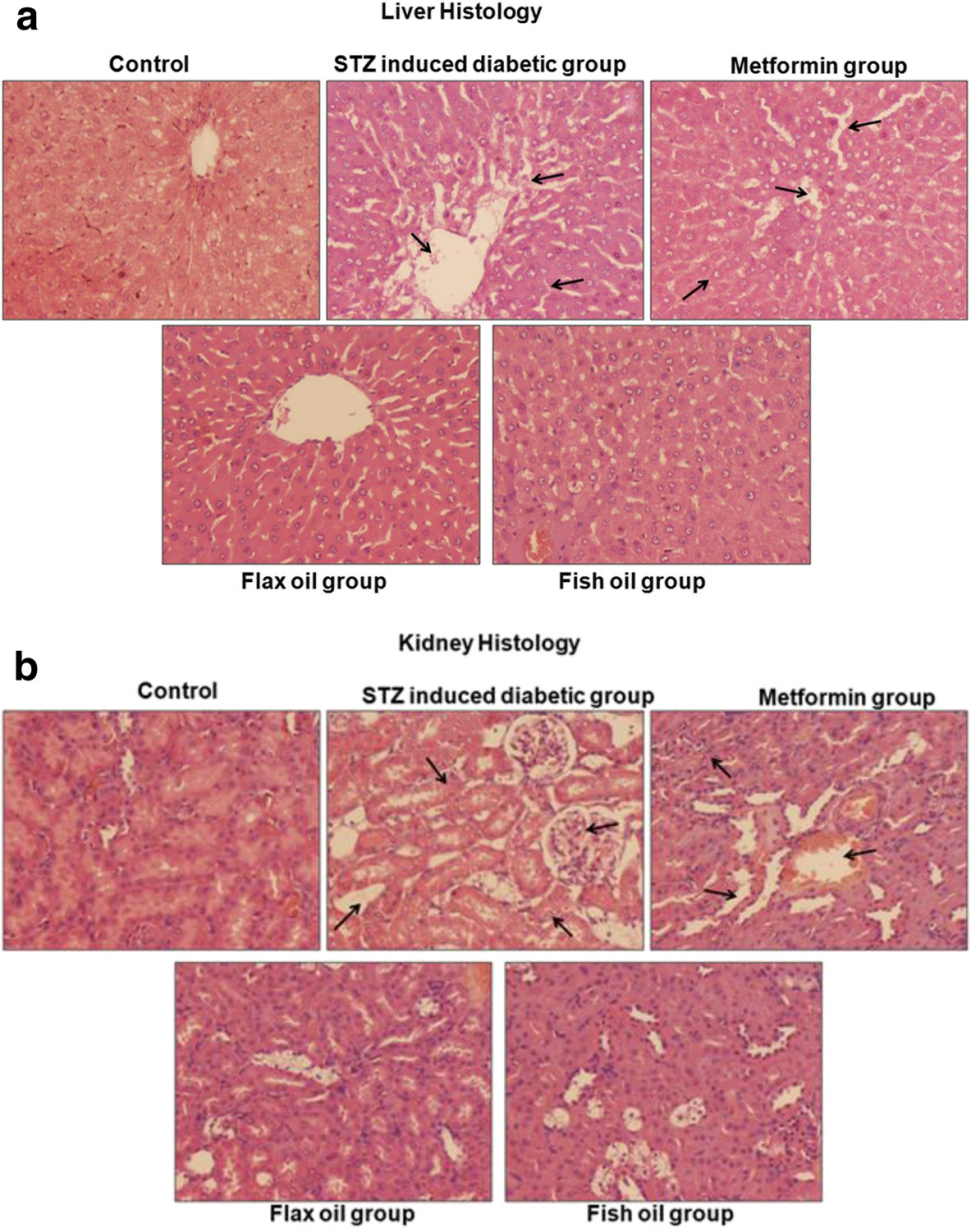
**a** Liver histology of control, STZ-induced diabetic, metformin-treated, and flax/fish oil-treated animals. Hematoxylin and eosin-stained cross sections of paraffin-embedded liver tissues of rats from the control and experimental groups (×40). Liver from the control group shows normal architecture. Sections of the liver from the STZ-induced diabetic group show severe destruction of hepatic cells, pathological calcification, hemorrhages, and mild mononuclear cells in the portal tracts. Liver from the metformin-treated group shows some destructive changes and congestion of some central vein. The liver histology of animals treated with flax oil and fish oil shows completely normal liver architecture without any anatomically detectable anomalies. **b** Kidney histology of healthy, STZ-induced diabetic, metformin-treated, and flax/fish oil-treated animals. Hematoxylin and eosin-stained cross sections of paraffin-embedded kidney tissues of rats from the control and experimental groups (×40). Kidney from the control group shows normal kidney architecture. Sections of kidney from the STZ diabetic group showed conjunction of glomerular capillary and blood vesicle. Some tubular epithelial cells show vacuolation and cloudy changes. Kidney from the metformin-treated group shows vacuolation of some tubular epithelial cells and conjunction of glomerular capillary. The liver histology of animals treated with flax and fish oil shows no considerable changes and show normal architecture

In the STZ-induced diabetic group, we observed some areas with pathological calcification in the partial triad focal hemorrhages and destruction of some bile ducts. It also showed destruction of hepatocytes, loss of hepatic lobules, and conjunction of the central vein. Surprisingly, the metformin group displayed destruction of some hepatocytes and congestion of the central vein. The flax and fish oil group displayed near-normal liver histology without any histological detectable anomalies (Fig. 3a).

### Renal function test markers

At D15, serum urea (*p* < 0.01) levels were higher in the STZ group as compared to those in the control. However, metformin and flax and fish oil did not show significant differences in renal function test markers at D15 and D30 (Table 4).

### Comparison of renal function test markers at D15 and D30 within the groups

There was no difference in the renal function test markers in the control, STZ, and metformin groups. The urea levels were higher (*p* < 0.01) in the flax oil group while total protein levels were lower (*p* < 0.05) in the fish oil group at D30 as compared to those at D15 (Table 5).

### Kidney histology

STZ group displayed blood vesicle conjunction, vacuolation of tubular epithelial cells, conjunction of glomerular capillary, cloudy change in many tubular cells, and some degeneration of the glomerulus. The metformin group displayed vacuolation of some tubular epithelial cells and conjunction of glomerular capillary. The flax oil and fish oil groups exhibited near-normal architecture and displayed significant recovery of the damage as compared to the STZ group (Fig. 3b).

## Discussion

The general characteristics of STZ-treated diabetic rats include low body weights and elevated blood glucose (Howarth et al. 2005). We observed a significant decrease in body weights and sustained hyperglycemia in nicotinamide-STZ-treated rats indicating successful induction of diabetes. In the present study, both metformin and omega-3 fatty acids could not normalize the glucose levels at D15 which remained higher till D30 of treatment. However, fish oil treatment lowered the glucose levels at D30 as compared to those at D15. It has been reported that metformin alone may not adequately control hyperglycemia (Salama et al. 2013) and omega-3 fatty acids do not directly affect glucose homeostasis (Woodman et al. 2002). However, several studies have reported hypoglycemic- and insulin-sensitizing effects of metformin (Maheshwari et al. 2014; Mohammadi et al. 2012; Erejuwa et al. 2011) and omega-3 fatty acids (Jangale et al. 2013) in the STZ-nicotinamide-induced diabetic rats. Consistent with our findings, metformin treatment at a dose 500 mg/kg for 8 weeks was not able to lower blood glucose in diabetic rats (Alhaider et al. 2011). These differences are probably due to variations in the duration of treatment or dose of metformin/omega-3 fatty acids which needs further investigation.

### Effect of n-3 PUFA intervention on lipid profile

Several reports have documented significant lipid abnormalities, like elevated cholesterol, triglyceride, LDL, and VLDL in diabetic rats (Nasrolahi et al. 2012)_._ We observed increase in the serum cholesterol, triglyceride, and VLDL levels in the STZ group indicating adverse effects of elevated glucose on lipid profile. However, long-term metformin treatment for 30 days reduced these levels. The beneficial lipid-lowering effects of metformin have been reported in diabetic rats (Nasrolahi et al. 2012). We have recently reported that long-term use of oral hypoglycemic agents significantly reduce triglyceride levels in diabetic men and LDL and HDL levels in diabetic women (Ghadge et al. 2014).

In the present study, flax oil and fish oil intervention normalized all lipid profile parameters at D30 as compared to those at D15. Several studies have reported triglyceride-lowering effects of dietary fish oil (Bremer et al. 2014; Hartweg et al. 2008) which have been primarily attributed to EPA and DHA (Skulas-Ray et al. 2011; Egert et al. 2009). The present study indicated activation of PPAR and modulation of SREBP1 in the animals treated with n-3 PUFA which are known to be the main mechanisms for a lipid-normalizing action of omega-3 fatty acids (Devarshi et al. 2013).

### Modulation of lipid metabolism genes by n-3 PUFA intervention

In the present study, STZ induction markedly reduced the expression of PPAR and increased SREBP1 expression which was restored by metformin as well as by fish oil (Fig. 2b). Metformin treatment is known to regulate incretin receptor axis via PPAR-dependent pathway in mice (Maida et al. 2011) and reduce SREBP expression by regulating AMP-activated protein kinase activity in rats (Zhou et al. 2001). Metformin has been shown to reduce fat content by decreasing the expression of SREBP1 and FAS (involved in fatty acid synthesis) in rat kidneys (Wang et al. 2006). A recent study reports reduction in PPAR expression and increase in SREBP expression in diabetic rats which was restored by dietary fish and flax oil supplementation (Devarshi et al. 2013). n-3 PUFA and their metabolites act as natural ligands for PPAR, promoting fatty acid oxidation and suppressing the transcription of lipogenic genes like FAS (Teran-Garcia et al. 2007).

ACSL, catalyzing the thioesterification of fatty acids, is a target of PPAR and is implicated in the pathogenesis of diabetes (Phillips et al. 2010). Recent report suggests that ACSL plays an important role in triglyceride synthesis (Yan et al. 2015). In the present study, STZ-treated animals showed significantly higher ACSL expression with high triglyceride levels. However, metformin and flax and fish oil interventions lowered the expression and consequently had significantly lower triglyceride levels. Metformin has been shown to lower ACSL expression and triglyceride levels in diabetic rats (Forcheron et al. 2009). Besides triglyceride synthesis by ACSL, MCAT, a mitochondrial protein, catalyzes transfer of CoA moiety to free thiol group on the acyl carrier protein in mitochondria, indicating its role in mitochondrial fatty acid synthesis (Zhang et al. 2003). Lower expression of MCAT along with ACSL has beneficial effects in normalizing the fatty acid and triglyceride levels (Li et al. 2009; Zhang et al. 2003). In the present study also, metformin and flax and fish oil showed reduced MCAT expression indicating lower fatty acid synthesis and normal lipid profile (Fig. 2b).

### Modulation of inflammatory cytokine genes by n-3 PUFA intervention

T2DM is a chronic inflammatory disease where transcription factor NFkβ activates the transcription of inflammatory cytokines like TNFα increasing the risk of secondary complications owing to their pro-atherogenic nature (Jagannathan-Bogdan et al. 2011). In the present study, NFkβ expression was higher in the STZ group which was significantly lowered by flax and fish oil (Fig. 2b). However, TNFα expression was not significantly altered in the STZ group and treatment groups. Hyperglycemia-induced NFkβ activation in ex vivo isolated PBMCs has been reported in type 1 diabetic patients (Hofmann et al. 1998). Metformin administration has also been shown to downregulate the expression of NFkβ and TNFα and ameliorate β-cell dysfunction (Liu et al. 2014) in diabetes. Recent review highlights the role of n-3 PUFA in reducing TNFα expression (Ellulu et al. 2015).

### Effect of n-3 PUFA intervention on liver function test markers

In the present study, SGPT and ALP levels were higher in the STZ group at D15 while ALP remained higher at D30 as compared to that in the control group. There are reports indicating hepatic dysfunction due to higher levels of liver enzymes such as SGPT, SGOT, and ALP in STZ-induced diabetic rats (Sajitha et al. 2012). We have recently reported higher serum SGPT and bilirubin levels in diabetic male subjects and SGPT levels in diabetic female subjects (Ghadge et al. 2015). The biochemical alterations in the hepatic function markers observed in the present study were also associated with destructive changes in hepatocytes and accumulation of lipid droplets.

In this study, metformin treatment did not affect other markers. In contrast, a study in diabetic rats treated with metformin is reported to have elevated bilirubin levels (Nasrolahi et al. 2012). Liver section analysis of these animals displayed destruction of some hepatocytes and congestion of the central vein. A recent study reported mild granular degeneration, mild swelling (narrow sinusoidal capillaries), and normal hepatic architecture in the metformin-treated diabetic rats (Motshakeri et al. 2014). Some case report studies also demonstrated that metformin induces hepatotoxicity in diabetic subjects raising concern about its efficacy and safety profile (Miralles-Linares et al. 2012; Cone et al. 2010).

Hepatoprotective effects of n-3 PUFA in chemically induced hepatotoxicity have been reported earlier from our laboratory (Chavan et al. 2013). In the present study, flax and fish oil intervention showed progressive effects in lowering ALP levels at D30 as compared to D15. Additionally, both of these groups exhibited normal hepatic architecture and displayed significant recovery of destructive changes.

### Effect of n-3 PUFA on renal function test markers

Serum urea levels were higher in the STZ group at D15 as compared to those in the control group. Although renal function test markers at D30 were comparable to those of the control group, STZ induction still affected kidney histology and are consistent with other reports (Ahmed et al. 2014; Zafar et al. 2009). The metformin group showed no significant changes in the renal function test markers against the STZ group. However, we observed vacuolation of tubular epithelial cells in some tubules and conjunction of glomerular capillary in metformin-treated group. Similar findings like moderate cellular hydropic degeneration, atrophied renal corpuscle, and mild congestion of glomerular capillaries were reported in metformin-treated diabetic rats (Motshakeri et al. 2014). Further, in the present study, fish oil treatment progressively lowered total protein levels at D30 as compared to those at D15. Flax and fish oil also showed normal kidney histology. Similarly, dietary flax and fish oil have shown to improve renal abnormalities in rats (Hassan et al. 2015; Velasquez et al. 2003).

## Conclusion

In conclusion, the treatment with metformin and flax/fish oil intervention in diabetic rats improves serum lipid profile and the expression of transcription factors modulating lipid metabolism such as PPAR-γ and SREBP1 and lowers atherogenic cytokine like NFkβ. In the present study, flax oil and/or fish oil exhibited hepatoprotective and nephroprotective effects under uncontrolled hyperglycemic states. The combination therapy of metformin and n-3 PUFA intervention is worth investigating in T2DM subjects.
